# Emphysema

**Published:** 1882-01-20

**Authors:** R. L. Hinton

**Affiliations:** Arkansas


### EMPHYSEMA.

BY R. L. HINTON, M. D., OF ARKANSAS.

I have just read in the August number of the RECORD, an article by Dr. James A. Low, of New York, entitled, "Emphysema and Bronchial Dilatation," in which he calls for practical suggestions as to treatment. An almost fac-simile case fell into my hands six months ago; told me that he had been afflicted about three years; that every physician who examined him said he had consumption, and that he had been under treatment for that disease all the time, but had been gradually growing worse. After a careful examination I told him he had emphysema of the lungs; that he not only didn't have consumption, but that one with his disease rarely, if ever, had consumption. His countenance brightened up wonderfully at this statement; said he felt as if a great burden had been rolled off him. I put him upon Tilden's malto-peptine, grs. x with pure linseed oil one tablespoonful before each meal; and iodide potash, grs. v, brom. potash, grs. x, in a tablespoonful of water and glycerine, equal parts, after each meal.

In a week he was much improved, and in four weeks said he thought he was about well, and went to work. He is a laboring man; has been doing pretty good work ever since, though he occasionally falls back upon his medicine.

I am not in the habit of writing for publication, and have written this very hurriedly upon reading the article referred to, with the desire to do no harm, if I do no good, and thinking it will, to some extent, answer the Doctor's request.

I prefer linseed oil to cod-liver oil because better tolerated by the stomach, more easily digested and fully as nutritious.

P. S.—I gave this man no other treatment, save 5 grs. of quinine every morning.

